# Cardiac magnetic resonance myocardial feature tracking correlates with natural radial strain and corresponds to inotropic stimulation

**DOI:** 10.1186/1532-429X-14-S1-O50

**Published:** 2012-02-01

**Authors:** Andreas Schuster, Shelby Kutty, Asif Padiyath, Victoria Parish, Paul Gribben, David A Danford, Marcus R Makowski, Boris Bigalke, Philipp B Beerbaum, Eike Nagel

**Affiliations:** 1 Imaging Sciences and Biomedical Engineering, KCL, London, UK; 2 Joint Division of Pediatric Cardiology, University of Nebraska/Creighton University, Children’s Hospital and Medical Center, Omaha, NE, USA; 3 Evelina Children’s Hospital, Department of Paediatric Cardiology, Guy's and St. Thomas' NHS Foundation Trust, London, UK; 4 Department of Radiology, Charite, Berlin, Germany

## Summary

We have demonstrated that cardiac magnetic resonance (CMR) myocardial feature tracking (FT) and natural radial strain correlate and correspond to inotropic stimulation. CMR-FT has the potential for quantitative wall motion assessment at rest and during dobutamine stress magnetic resonance (DSMR) imaging.

## Background

CMR-FT is a recently introduced technique for tissue voxel motion tracking on standard steady-state free precession (SSFP) images to derive radial myocardial mechanics. CMR-FT has the potential to facilitate DSMR analysis however has not yet been compared to external reference standards (with stress) such as SSFP derived natural radial strain.

## Methods

10 healthy subjects were studied at 1.5 Tesla. LV short-axis radial strain ErrSAX was derived from SSFP cine images using dedicated CMR-FT software (Diogenes MRI prototype, Tomtec, Germany) at rest and during dobutamine stress (10 and 20 μg * kg-1* min-1). Natural radial strain values (loge [End-systolic wall thickness/end-diastolic wall thickness]) were calculated in identical segments as analysed for ErrSAX using commercially available software (Philips View Forum, The Netherlands). 95% confidence intervals (CI) of the difference and p-values were calculated to compare the 2 techniques.

## Results

In all volunteers strain parameters could be derived from the SSFP images at rest and stress. ErrSAX values showed significantly increased contraction with DSMR (rest: 19.6±14.6; 10 μg: 31.8±20.9; 20 μg: 42.4±25.5, p<0.05). Natural radial strain values increased with dobutamine (rest: 24±8.9; 10 μg: 36.5±8.9; 20 μg: 44.2±8.5, p<0.05).

**Figure 1:**
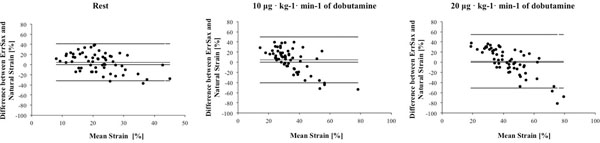
Bland Altman Plots showing the relationship between ErrSAX and natural radial strain. ErrSAX = left ventricular short-axis radial strain.

There was reasonable agreement between mean ErrSAX and natural radial strain at rest and with dobutamine stress (figure 1 and table 1) as determined by 95% CI of the difference.

**Table 1:** 

Parameter	Ventricle	Mean	CI (95%)	p-value
Rest	ErrSAX	19.6	15.8-23.4	0.07
		
	Natural Strain	24	21.7-26.4	

10 μg * kg-1* min-1 of dobutamine	ErrSAX	31.8	26.9-37.9	0.12
		
	Natural Strain	36.5	34.2-38.8	

20 μg * kg-1* min-1 of dobutamine	ErrSAX	42.4	35.8-48.9	0.59
		
	Natural Strain	44.2	42-46.4	

The table shows variability between CMR-FT radial strain parameters (Err) and natural radial strain of the LV derived from a short-axis view at rest and with dobutamine stress. 95% Confidence Intervalls of the difference and p-values are given to accurately determine individual variabilities. ErrSAX = left ventricular short-axis radial strain.

## Conclusions

CMR-FT correlates with natural radial strain derived from SSFP cine imaging. Both measures correspond to inotropic stimulation. CMR-FT holds promise of easy and fast quantification of wall mechanics and strain.

## Funding

AS receives grant support from the British Heart Foundation (BHF) (RE/08/003 and FS/10/029/28253) and the Biomedical Research Centre (BRC-CTF 196). SK receives grant support from the American College of Cardiology Foundation, the Edna Ittner Pediatric Foundation, and the Children’s Hospital and Medical Center Foundation.

